# Portable, and ultrasensitive HR-HPV tests based on nucleic acid biosensors

**DOI:** 10.3389/fcimb.2024.1357090

**Published:** 2024-02-28

**Authors:** Chang Ma, Minhong Zou, Ning Xu, Yan Liu, Yuru Wang

**Affiliations:** ^1^ College of Public Health, Jilin Medical University, Jilin, China; ^2^ State Key Laboratory for Diagnosis and Treatment of Severe Zoonotic Infectious Diseases, Key Laboratory for Zoonosis Research of the Ministry of Education, Institute of Zoonosis, and College of Veterinary Medicine, Jilin University, Changchun, China

**Keywords:** high-risk human papillomavirus, nucleic acid amplification, biosensor, point-of-care test, molecular detection

## Abstract

Cervical cancer is the third most common cancer threatening women’s health globally, and high-risk human papillomavirus (HR-HPV) infection is the main cause of cervical cancer worldwide. Given the recurrent nature of HR-HPV infection, accurate screening is essential for its control. Since the commonly used polymerase chain reaction (PCR) technique is limited by professional equipment and personnel, convenient and ultrasensitive detection methods for HR-HPV are still highly needed. As new molecular detection methods, nucleic acid amplification-based biosensors have the advantages of high sensitivity, rapid operation, and portability, which are helpful for point-of-care testing in rural and remote areas. This review summarized nucleic acid biosensors for HR-HPV screening based on a variety of nucleic acid amplification strategies involved in improved PCR, loop-mediated isothermal amplification, recombinase polymerase amplification, hybridization chain reaction, catalyzed hairpin assembly, and CRISPR/Cas systems. In combination with microfluidic technology, lateral flow assays, electrochemical analysis and other sensing technologies, HR-HPV nucleic acid biosensors have the advantages of high throughput, short response time, high sensitivity and easy operation in the field. Although there are still shortcomings, such as high cost and poor reproducibility, this approach will be suitable for on-site screening of HR-HPV infection or cervical cancer and for auxiliary clinical diagnosis in complex environments and poor areas in the future.

## 1 Introduction

Cervical cancer is the third most prevalent cancer in women (Hohn et al., 2021; Serrano et al., 2022) and is a major cause of death worldwide (Siegel et al., 2022), especially in developing countries with poor medical conditions (Small et al., 2017). High-risk human papillomavirus (HR-HPV) is the most common sexually transmitted infection (Boon et al., 2022) and is the primary cause of cervical cancer (Schiffman et al., 2007; Perkins et al., 2020; Arbyn et al., 2021). In general, infection with regular HPV can be controlled by the immune system, but persistent infection and reinfection with HR-HPV can lead to precancerous lesions. HR-HPV screening is not widely available for use in developing countries because of the high mortality and morbidity rates of cervical cancer (Ronco et al., 2014; Szymonowicz and Chen, 2020). Persistent infection and reinfection with HR-HPV can lead to precancerous lesions and other malignant cancers (Berman and Schiller, 2017; Yu et al., 2022). Nearly all cervical cancer cases are caused by persistent infection with HR-HPV (Depuydt et al., 2012b; Fontham et al., 2020), which has a double-stranded DNA genome without an envelope (Cheng et al., 1995; de Villiers et al., 2004; Doorbar et al., 2012; Kombe Kombe et al., 2020; McBride, 2022). HPV types 16 and 18 cause approximately 70% of cervical cancer and precancerous lesions (Moody and Laimins, 2010; Yousefi et al., 2021; Moch et al., 2022) and are the most common HR-HPV types (Boulet et al., 2008; Lizano et al., 2009; Cohen et al., 2019; Johnson et al., 2020; Park, 2020). The World Health Organization (WHO) recommends nucleic acid amplification tests (NAATs), such as polymerase chain reaction (PCR), as the preferred method for detecting HPV (Abu-Rustum et al., 2020; World Health, 2022) (Al Roomy et al., 2023). At present, PCR is the most common detection method (Schmittgen and Livak, 2008; Depuydt et al., 2012a; Organization et al., 2017; Piyathilake et al., 2022). However, PCR is usually limited to exclusive procedures performed in large centralized clinical laboratories, complex operations, time-consuming procedures, specialized equipment and professional personnel. These disadvantages restrict the rapid and accurate detection of cervical cancer. Therefore, convenient and ultrasensitive detection of HR-HPV is still highly desirable, especially in poor countries and rural areas (Manos et al., 1990; Bhatla and Singhal, 2020). Recent studies have focused on nucleic acid amplification with biosensors, which are able to achieve point-of-care test (POCT) results and assist in the diagnosis of cervical cancer (Wang et al., 2002; Pagliusi and Garland, 2007; Arbyn et al., 2012). Major advances in POCT have led to enormous opportunities for patient stratification and accessible monitoring, especially in rural and remote areas (Eun and Perkins, 2020).

**Figure 1:**
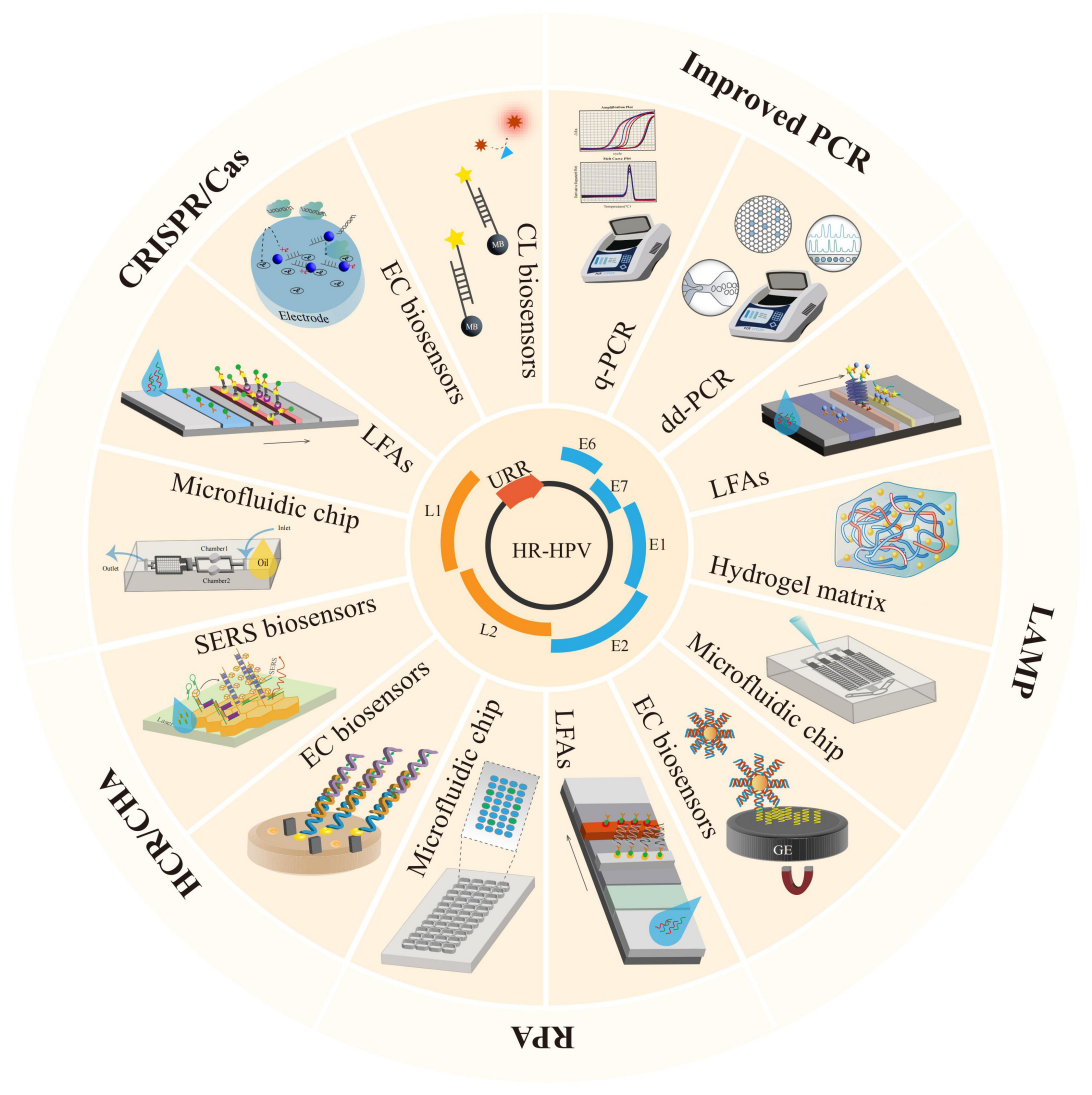
Schematic illustration of various categories of HR-HPV nucleic acid biosensors.

NA-based biosensors typically consist of nanoscale signal transduction, with nanoparticles filling the gap between the transducer and the bioreceptor (Malik et al., 2023). The use of nanomaterials significantly improves the sensitivity of the assay. Various types of nanostructures can be used to improve the performance and efficiency of sensors in their structures (Huang et al., 2021). Including nanoscale droplets (droplet digital PCR), nanoparticles (gold nanoparticles, ZnO, zirconium-based metal), nanotubes, and nanocomposites. In addition to NA-based biosensors, other used diagnostic methods for HR-HPV infection include the FDA-approved PCR-based amplification assay Cobas 4800 HPV test (Isidean et al., 2014; Salazar et al., 2019) and BD Onclarity (Latsuzbaia et al., 2022). For example, Hybrid Capture II (Serour et al., 2017) uses a gene hybridization signal amplifier to amplify the signal captured by the antibody and detect the chemiluminescent signal. In contrast, Cervista is a cleavage-based enzymatic signal amplification assay for the detection of high-risk HPV (Guo et al., 2014). Biosensors combining nanomaterials and nucleic acid testing can effectively enable POCT. Here, we summarize the latest progress in HR-HPV detection based on nucleic acid amplification combined with nano biosensors (Boulet et al., 2008), which include microfluidic technology, lateral flow assays (LFAs), electrochemical (EC) analysis, and other sensing technologies, providing references for the rapid development of POCT in remote areas (**Figure 1**).

## 2 Improved PCR-related HR-HPV detection methods

As one of the most widely used tests for detecting HR-HPV, PCR is important for primary screening (Arbyn et al., 2012) and diagnosis (Perkins et al., 2023), and the ASCCP recommends it for follow-up use after treatment. Quantitative PCR (q-PCR) is a high-throughput method for HPV detection, quantitative analysis, and viral typing (Wang et al., 2002; Thammaiah et al., 2018). Moreover, the viral load can be determined or HPV status can be integrated to support cervical cancer screening (Schmittgen and Livak, 2008; Micalessi et al., 2011; Organization et al., 2017). Compared the improved PCR with the traditional PCR assay test (Snijders et al., 1990; de Roda Husman et al., 1995) in **Table 1**, has lower detection limits, higher sensitivity, and higher specificity than conventional PCR, however, they both rely on large instruments. Analysis of specimens from HPV positive clinical patients using the q-PCR method enables further confirmation of the viral load. Compared with traditional PCR, q-PCR reduces clinical false-positives and achieves high sensitivity (0.46 copies/cell) by identifying BSGP5+/6+-PCR/MPG of HPV-16 (Schmitt et al., 2013). Multiplexed q-PCR was used to target the E6 and E7 genes of HPV-16 and HPV-18, with a sensitivity of up to 10 copies/µl (Thammaiah et al., 2018; Bordigoni et al., 2021). qPCR using GP5+/6+ primers quantified the genome with a dynamic detection range of 1.91 to 3.4 million and a limit of detection (LOD) of approximately 200 (Shen-Gunther and Yu, 2011). Moreover, the cost of q-PCR is less than 10 dollars to achieve high-throughput testing and auxiliary histological and cytological clinical diagnosis. In addition, q-PCR based on internal probes is a fast, easy, specific and reproducible method that requires minimal DNA and does not require specialized, analysis-specific laboratory instrumentation.

**Table 1:** The performance comparison of PCR-based methods in HR-HPV detection.

Amplification Technology	Transduction	Times (min)	LOD	POCT	Cost	Instrument	Accuracy (%)	Specificity (%)	Sensitivity (%)	PPV (%)	NPV (%)	Ref.
Traditional PCR	Fluorescent	-*	500 copies/cell	No	Expensive	Heavy	-*	-*	43-89.4%	-*	-*	(Snijders et al., 1990; de Roda Husman et al., 1995)
q-PCR	Fluorescent	-*	0.46 copies/cell	No	Expensive	Heavy	95%	95.7	100	95.7	94.4	(Schmitt et al., 2013)
	Fluorescent	-*	10 copies/µl	No	Expensive	Heavy	-*	≥95	100	-*	-*	(Bordigoni et al., 2021)
dd-PCR	Fluorescent	-*	34 copies/cell	No	Expensive	Heavy	-*	-*	96.8	-*	-*	(Okada et al., 2019)
	Fluorescent	300	100 copy/reaction	No	Expensive	Heavy	99.9–100%	100	-*	-*	-*	(Rotondo et al., 2020)
	Fluorescent	-*	1000 copies/mL	No	Expensive	Heavy	-*	97.8	76.0-100	-*	-*	(Galati et al., 2022)
	Fluorescent	-*	1.6 copies/reaction	No	Expensive	Heavy		100		-*	-*	(G. and Helenius, 2017)

NPV, Negative predictive value.

PPV, Positive predictive value.

-*, No test.

Droplet digital PCR (dd-PCR) was first proposed in 1990 to disperse PCR systems into nanoscale droplets for segmented amplification and precise quantification (Vogelstein and Kinzler, 1999). The absolute concentration of the sample can be calculated using the Poisson distribution (Hindson et al., 2013; Malin et al., 2021) without the need for a standard curve, which greatly improves the relative expansion certainty of the copy number concentration (Pinheiro et al., 2011). The sensitivity of dd-PCR for E6 and E7 HR-HPV testing is significantly greater than that of q-PCR (Okada et al., 2019; Rotondo et al., 2020; Galati et al., 2022; Mattox et al., 2022). Both tissue samples soaked in formalin-fixed paraffin-embedded tissue and target DNA extracted from liquid-based cytology can be used to detect HR-HPV, with an LOD of 1.6 for HPV-16 and 2.8 for HPV-18 (G. and Helenius, 2017; Stevenson et al., 2020). By combining digital analysis and 96-well workflows, dd-PCR can be used to construct high-throughput assays with more than 2 million reactions (Hindson et al., 2011). Nanoscale droplets generated by a stepped emulsification nozzle array self-assemble into a single-layer array in a U-shaped chamber (Hou et al., 2023). The portable detection of dd-PCR can be achieved by using a cationic microfluidics chip droplet dispersion system (Nie et al., 2019; Chen et al., 2022). Thus, initial diagnosis of HPV-associated lesions by HPV type-specific testing can help in the management of patients. Compared with the traditional PCR, the improved PCR assays have higher sensitivity and specificity, and the required sample volume and the limit of detection are much lower than the PCR.

## 3 Isothermal nucleic acid amplification related HR-HPV detection methods

Isothermal nucleic acid amplification has a few advantages, such as its speed, efficiency, portability, and strong specificity. It can detect nucleic acids or the expression of genes in living cells (Yan et al., 2014). Constant temperature amplification can be used to diagnose pathogens by amplifying nucleic acids with a short signal reading time within a few hours (Gill and Ghaemi, 2008). These methods can be classified into enzyme-based methods and enzyme-free methods. Enzyme-based methods include loop-mediated isothermal amplification (LAMP) and recombinase polymerase amplification (RPA), while enzyme-free methods include hybridization chain reaction (HCR) and catalyzed hairpin assembly (CHA).

### 3.1 LAMP-related HR-HPV detection methods

**Figure 2:**
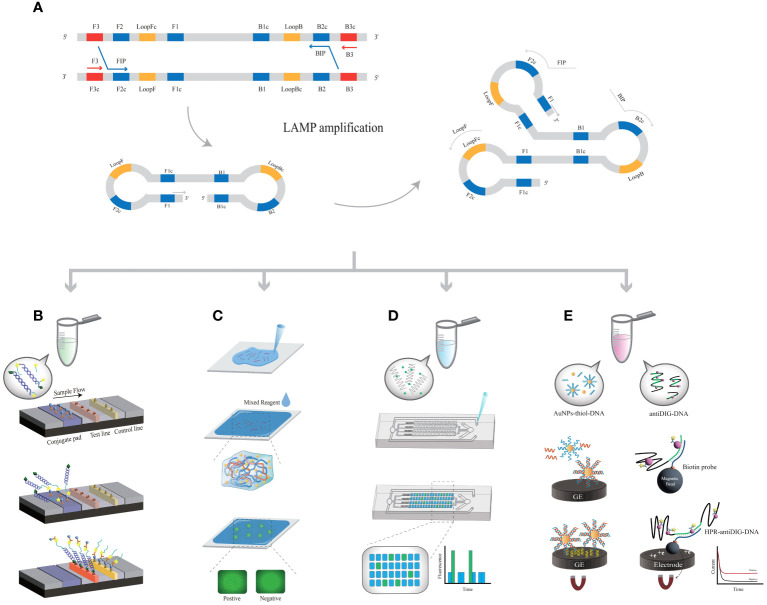
The principle of LAMP-related biosensors. **(A)** The principle of LAMP amplification. **(B)** LAMP-based assays coupled with LFA; each probe is labeled with fluorescein isothiocyanate. **(C)** The LAMP product is covalently linked to the hydrogel matrix. **(D)** LAMP products are added to microfluidic chips. **(E)** AuNPs modified with DNA or anti-DIG DNA capture LAMP products, further bonding with metal electrodes.

In 2000, Notomi (Notomi et al., 2000) proposed the use of specially designed primers and *Bst* DNA polymerase, which can replace strands by designing primers in six regions of the DNA double strand to form a neck loop structure (**Figure 2A**). Specific nucleic acid sequences can be amplified by repeatedly extending the neck loop structure upstream (FIP) and downstream (BIP) (Livingstone et al., 2016). In this way, 10^9^ specific nucleic acid amplifications at constant temperature can be achieved (approximately 60 °C for 45 min) (Notomi et al., 2015). In contrast to traditional signal reading methods such as turbidity detection and fluorescent colorimetric detection (Rohatensky et al., 2018; Landaverde et al., 2020; Yu et al., 2020), agarose gel electrophoresis and LAMP-based biosensors can be used to test multiple high-risk HPV genes conveniently and quickly (Zhang et al., 2022) in combination with flow chromatography test paper, 3D printing (Saetiew et al., 2011), microfluidics or smartphone reading systems.

LAMP was used in conjunction with a LFAs (Landaverde et al., 2020), and the HPV-16 and HPV-18 probes were labeled with fluorescein isothiocyanate (FITC) (**Figure 2B**). LFA was performed using amplified products labeled with biotin; this method allows for a sample-to-result detection time of less than 45 min, with an LOD of 1×10^1^ copies/μL. Compared with the traditional PCR, LAMP-LFA can complete detection in a shorter time with no relying on large equipment, but its sensitivity and specificity are slightly lower than the standard, and it is prone to contamination during the reaction (Landaverde et al., 2020). The FIP primer in the LAMP system was changed to a thiol group (Wang et al., 2022) so that the product was covalently linked to the hydrogel matrix (**Figure 2C**). After the hydrogel becomes solid, LAMP reaction is carried out *in-situ*, and the combination of the amplification product and DNA dye is limited in the microstructure of the gel, forming bright spots on HPV infected cervical cells. The results were recorded using a fluorescence microscope. In point-of-care testing, using a smartphone to take photos can record and analyze fluorescence points, and ultimately obtain detection results. The *in-situ* hydrogel methods associated with LAMP technology are low cost and allow for simultaneous large-scale detection, although only one type of HPV can be detected in one test. The bright fluorescent spots generated by amplification can be directly observed in the hydrogel with a short signal reading time. The hydrogel matrix effectively prevents cross reactions between two adjacent cells. The test could be completed within 30 min with an LOD of 100 copies/time, which is consistent with the gold standard. At the same time, point-of-care can also be used. Although the samples need to be processed by heat fixation, this method saves time by eliminating the cumbersome operation of large instruments and has the potential to be used in remote areas or large-scale screening.

The combination of LAMP with microfluidic digital chips can meet the demands of grassroots laboratories or on-site POCT, achieving widespread promotion and application (Yu et al., 2020). The microfluidics chip was prepared by combining hot temperature vacuum-treated polydimethylsiloxane. The oligonucleotides were immobilized on a microfluidic device, and the reaction process was ensured to be complete (**Figure 2D**). Combined with microfluidics, color visualization of HR-HPV can be achieved (Ho et al., 2018), with an LOD of 200 copies/test. When MnCl_2_ and calcein were added to the reaction system, the color was increased by the two byproducts, PPi4- and H^+^ ions, of the LAMP reaction. Using smartphones, the viral load was recorded and calculated (Yin et al., 2019), and 41 reaction samples were detected simultaneously (65°C, 90 min), with an LOD of 5-50 copies/μL (Wang et al., 2021). Introducing microfluidic chips into LAMP amplification can achieve portable HPV detection. Meanwhile, microfluidic sealed chips can effectively avoid the generation of aerosols, thereby improving sensitivity, accuracy, and specificity. This simple, multi-link, quantitative, CRISPR based detection platform is compatible with various nucleic acid biomarkers and has great potential in POCT diagnosis.

The combination of reverse transcription (RT-LAMP) and EC biosensors can achieve extremely sensitive detection of HPV (Prakrankamanant et al., 2013). When gold nanoparticles (AuNPs) are thiol-modified and immobilized on DNA, the amplification products are captured by DNA probes to form AuNP nanocomposites (**Figure 2E**). Many nanocomposites labeled with biotin were captured on graphite electrodes, with an LOD of 100 copies/μL (63 °C, 45 min). A validated nucleic acid test standard can be achieved in a short time with good sensitivity and specificity for future clinical diagnosis. LAMP products were captured by an anti-digoxin antibody (anti-DIG) labeled with free horseradish peroxidase (HRP) and then hybridized with streptavidin magnetic beads (MBs). This signal can be measured by enzyme catalysis on a carbon electrode chip (Bartosik et al., 2018; Yang et al., 2021). Based on the electric current applied during the amplification process, LAMP results can be obtained within 2 hours with an LOD of 10 cells (Izadi et al., 2021). The EC biosensor has the characteristics of ultra sensitivity, wide linear range, and high selectivity, which is of great significance for the early diagnosis and primary screening of HPV. However, it also faces drawbacks such as complex separation and the need for experimental equipment. A quartz crystal microbalance is a nonlabeled sensor that can be combined with LAMP and quartz crystal microbalance sensor technology (Prakrankamanant et al., 2013). The biotin-labeled LAMP products bind specifically and are captured on the quartz crystal microbalance surface, and the LOD of the biosensor was 100 copies/μL (30 min, 34.073.6 Hz). Compared to the portable and visual detection methods mentioned earlier, the current EC biosensors in the field of HR-HPV detection still face problems of complex operation and susceptibility to environmental interference, which urgently need to be addressed. The temperature of LAMP-based biosensors is significantly lower than that of PCR, which makes the amplification process independent of thermal cycling devices possible at the point-of-care (**Table 2**).

**Table 2:** The performance comparison of LAMP-based methods in HR-HPV detection.

Amplification Technology	Transduction	Times (min)	LOD	POCT	Cost	Instrument	Accuracy (%)	Specificity (%)	Sensitivity (%)	PPV (%)	NPV (%)	Ref.
LAMP	LFA	45	1×10^1^ copies/μL	Yes	-*	Portable	70.83	89	60	90	57	(Landaverde et al., 2020)
	*In situ* hydrogel	30	100 copies/time	Yes	Low-cost	Portable	100	100	100	100	100	(Wang et al., 2022)
	Microfluidic	110	10 fg/μL	Yes	-*	Portable	-*	-*	-*	-*	-*	(Yu et al., 2020)
		120	200 copies/test	Yes	Low-cost	Portable	94.4-96.5	100	83.3-92.9	86.7	95	(Ho et al., 2018)
		90	5-50 copies/μL	Yes	Low-cost	Portable	100	100	100	100	100	(Wang et al., 2021)
	EC	150	0.7 ng	Yes	Low-cos	Portable	100	100	100	-*	-*	(Bartosik et al., 2018)
		120	10 cells	Yes	Low-cost	Portable	-*	81.82-94.12	100	66.67	100	(Izadi et al., 2021)
		30	100 copies/μL	Yes	Low-cost	Portable	-*	100	90.5	85.7	100	(Prakrankamanant et al., 2013)
		45	100 copies/μL	Yes	Low-cost	Portable	100	100	100	-*	-*	(Yang et al., 2021)
	CRISPR/Cas12a-LFA	70	3.1×10^-21^ M	Yes	Low-cost	Portable	-*	100	-*	-*	-*	(Mukama et al., 2020)

NPV, Negative predictive value.

PPV, Positive predictive value.

-*, No test.

### 3.2 RPA-related HR-HPV detection methods

**Figure 3:**
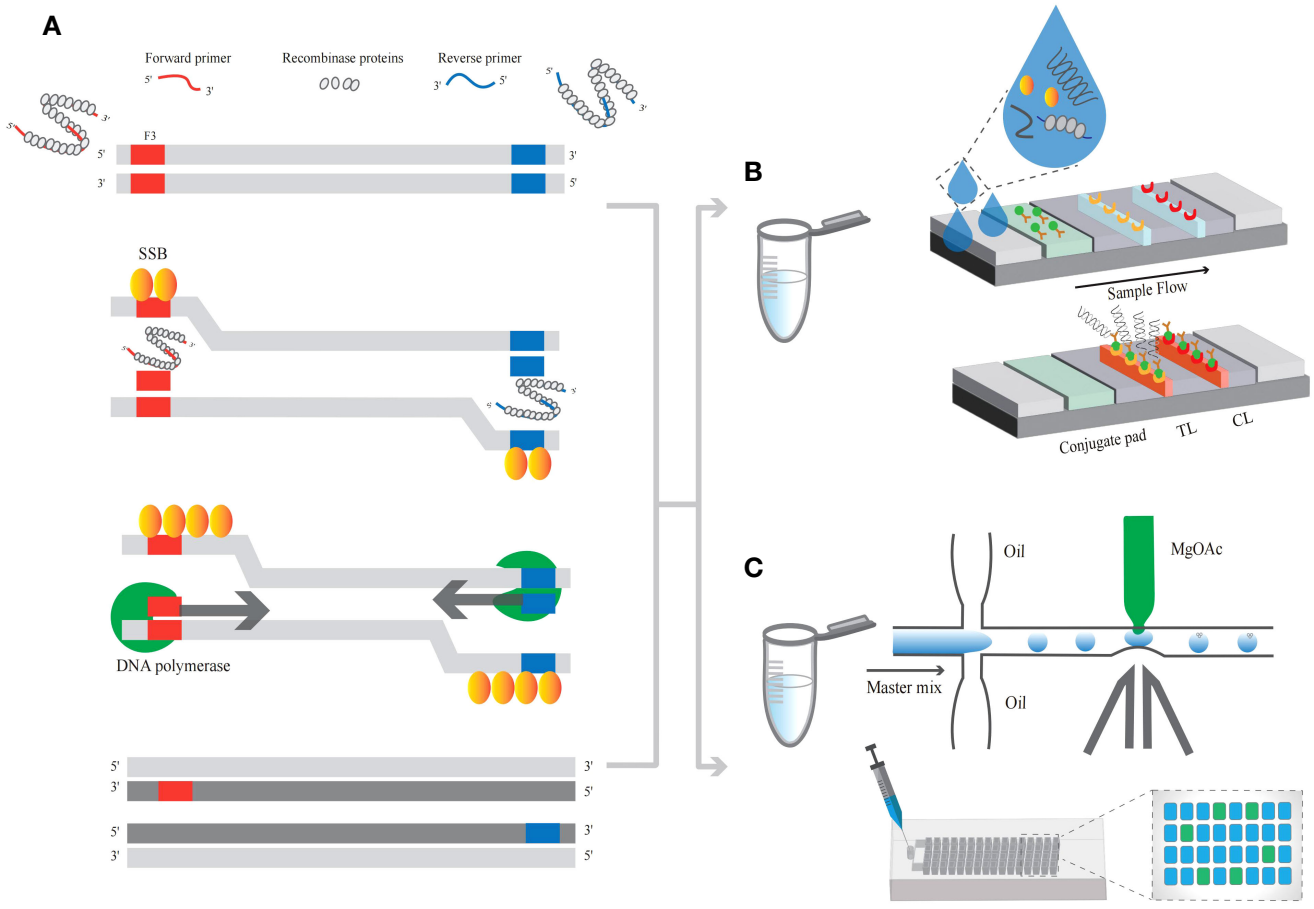
The principle of RPA-related detection. **(A)** The principle of RPA amplification. **(B)** RPA-based assays coupled with LFA. The presence of two red lines indicates a positive result. **(C)** RPA products emulsified into droplets accompanied by MgOAc injection. The fluorescence signals generated after amplification were analyzed via Poisson statistics.

Compared with LAMP technology, which is limited by a 55-65°C heating temperature, RPA can achieve nucleic acid amplification at lower temperatures (25-44°C). RPA technology relies on three proteins (**Figure 3A**): recombinase proteins, which can bind single-strand DNA (ss-DNA), single-strand DNA-binding protein (SSB) and strand replacement DNA polymerase (James and Macdonald, 2015; Bergeron et al., 2016). First, the RecA recombinase protein complex of *Escherichia coli* was used to introduce specific primers to the target site, and the amplification reaction was started by chain displacement polymerase. Recombinase proteins bind to primers to form protein−;DNA complexes, after which homologous sequences are searched for in double-stranded DNA (ds-DNA). Once the primers locate the homologous sequence, a chain exchange reaction occurs to form and initiate DNA synthesis, exponentially amplifying the target region on the template (Piepenburg et al., 2006; Li et al., 2018; Lobato and O'Sullivan, 2018).

Amplification products linking biotin-digoxin were added to the LFA crossflow pad by combining RPA with LFA (**Figure 3B**). Biotin-digoxin and ds-DNA products are captured by streptavidin immobilized on the assay line (Wang et al., 2020). A colloidal gold-labeled anti-DIG monoclonal antibody was used for color observation, with an LOD of 10 copies/μL (37 °C for 60 min), but HR-HPV typing could not be performed (Ma et al., 2019). The use of water-soluble graphene oxide and SAMRS primers significantly improved the specificity of RPA detection (Wang et al., 2020). In addition, the combination of LFA further simplified the detection of Nano RPA, which is an excellent point-of-care assay in the monitoring of disease without time-consuming or expensive procedures and equipment. After mature application, the cost of each detection can be controlled within 8 dollars, which will be suitable for areas with low-resources and poor medical environments. Simplified primer design and primer optimization using graphite oxide and self-avoidance molecular recognition systems. RPA specificity was improved by using a single labeled primer that binds to the LFA. A microfluidics microinjector, which consists of a flow-focusing droplet generator with a picoinjector coupled downstream (Cui et al., 2022), is used to detect drop digital recombination polymerase amplification (dd-RPA) (**Figure 3C**). The reaction initiator MgOAc was added to the droplet (RPA mixture) to control the unlocking of the digital reaction. The target DNA is amplified and bound to the T7 transcript, which is subsequently converted to RNA. Add 2μ the target samples separately to the reaction system and mix them as the aqueous phase generated by droplets. MgOAc, as an injection phase, will activate droplets containing target DNA upon addition. The magnified target can be detected using fluorescence sensing methods. The results of verifying clinical specimens are 100% consistent with those of PCR. RPA-microfluidic is suitable for POCT and remote areas. The integration and simplification methods implemented by microfluidic technology have opened up new avenues for advancing precise digital quantification tools in microfluidic technology, which may be the trend for *in vitro* detection. CRISPR/Cas13a cutting was used to detect fluorescence, and the results were calculated by the Poisson distribution, with an LOD of 10 copies/μL (37 °C, 30 min).

All of the amplification methods mentioned above require the participation of biological enzymes. Active components such as biological enzymes and proteins are prone to interference from various natural conditions, such as temperature, pH, perishability, poor stability, degradability, etc., resulting in false-positives, poor repeatability and a high reaction background (Gill and Ghaemi, 2008). The technique of enzyme-free amplification is gradually being applied to the field of nucleic acid amplification. The principle of enzyme-free amplification is based mainly on the self-assembly of DNA fragments that can convert target genes into hybrid products or on the toehold-mediated strand displacement (TMSD) system (Zhang and Seelig, 2011; Yang et al., 2015). The structural domain at the end of single-stranded DNA, called the sticky end (toehold), can be continuously opened by two hairpin structures. Thermodynamically, the interaction force between two strands is blocked, and two single strands interact with each other (Yin et al., 2008). The toehold exchange process was introduced as a method for designing a fast reversible chain single displacement reaction (Simmel et al., 2019). HCR and CHA require only two nucleic acid strands and one initiator strand for amplification, which is highly efficient and sensitive.

## 4 Enzyme-free amplification-related HR-HPV detection methods

### 4.1 HCR assays

**Figure 4:**
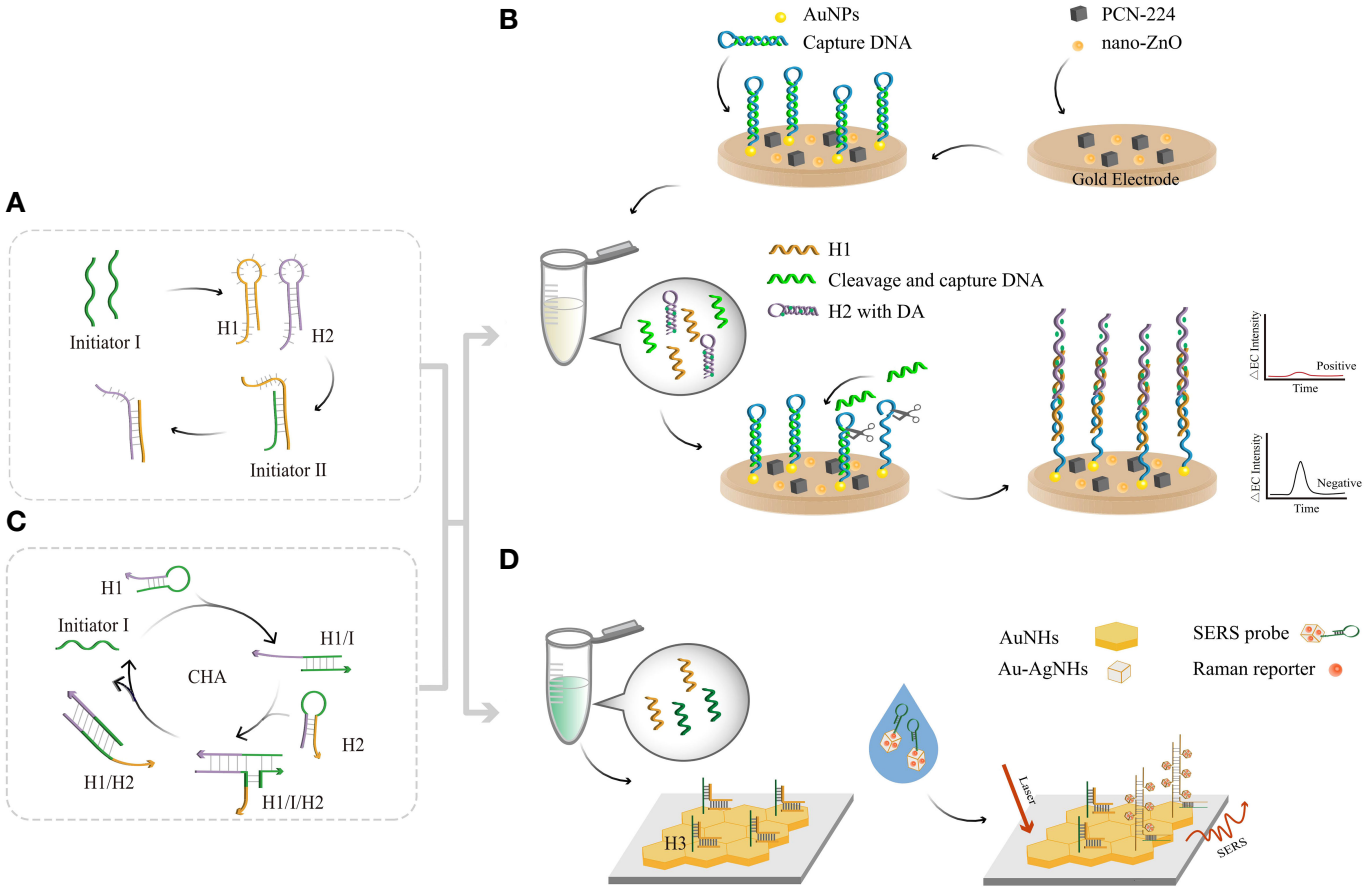
The principle of enzyme-free amplification-related detection. **(A)** The principle of HCR amplification. **(B)** HCR-based assays coupled with an EC biosensor. The HCR product combines with AuNPs and then continues to amplify through exonucleases. Reduction of electrical signals by connecting a large number of quenching probes to the electrodes. **(C)** The principle of CHA amplification. **(D)** SERS sensing strategy via cascade CHA and HCR. Moreover, a large number of Au-AgNCs and Raman reporters clustered other substrates, enhancing the SERS signals with each other.

In 2004, Dirks and Pierce proposed the HCR (Dirks and Pierce, 2004; Bi et al., 2017), in which DNA can perform both recognition and signal amplification by binding to substrates. HCR has the advantages of being enzyme free and isothermal and has a controllable synthesis route and good biocompatibility. Two types of hair clips (H1 and H2) captured by kinetic traps can coexist stably without the addition of initiator I (**Figure 4A**). Upon introduction, the H1 chain is opened, and the H1 binds to the sticky end of H1 via the TMSD reaction. By opening the chain of the upstream hairpin structure of H1, the newly exposed upstream domain of H1 can serve as another initiator to complement and bind to the viscous end of H2. By opening the hairpin structure upstream of H2, initiator I can continue to open the H1 chain and amplify the target DNA until H1 and H2 are exhausted. The molecular weight of the HCR product is inversely proportional to the amount of initiator. Di Wu et al. (Wu et al., 2022) developed an electrochemiluminescence (ECL) biosensor platform using HCR coupled with nano-ZnO, zirconium-based metal (PCN-224) and polyacrylamide (PAM) (**Figure 4B**). These materials were modified to be the substrate material for EC biosensing, which improved the signal detected. AuNPs covalently bind to the electrode surface, and the capture DNA combines with them. Amplification of target DNA quantities by exonuclease-based DNA cyclic cleavage and generation of large quantities of cleaved and captured DNA followed by HCR at the electrode (Cui et al., 2020). Detection of the target EC signal was achieved by “signaling off”. The values of ΔEC and HR-HPV showed a logarithmic correlation from 1 fM to 1 nM, with an LOD of 1.41 aM. Construction of EC biosensors based on differences in electrostatic interactions by combining the HCR with EC electrostatic interaction sensors (Lu et al., 2022).

### 4.2 CHA assays

Typically, the CHA reaction involves the use of two complementary DNA strands prepared as stable hairpin structures (**Figure 4C**). In 2008, Yin and Pierce designed two complementary DNA hairpin structures, H1 and H2 (Yin et al., 2008), and a single-stranded oligonucleotide initiator, I (Liu et al., 2019). First, initiator I recognizes the toehold of H1 through a specific action to form the H1/I complex. Subsequently, the exposed H1 toehold recognizes the toehold of H2, producing unstable H1, I, and H2 complexes (McConnell et al., 2021). The newly released H2 leads to branch migration and dissociation from H1/I. This disassembly of hairpin DNA structures enables 100-fold H1/H2 double-stranded amplification in a short period of time. Dan Lu et al. used a cascade signal amplification technique based on CHA and HCR (Dan et al., 2022) in conjunction with a surface-enhanced Raman scattering (SERS) biosensor to design a method to detect HR-HPV. This method provides a signal enhancement of 6-14 orders of magnitude compared to that of conventional Raman signals (**Figure 4D**) and uses HR-HPV conjugates as triggers to self-assemble hairpin DNA into CHA products. When the product is dropped on the Au nanohexagon (AuNH) array, the H3 chain is opened, while the SERS probe is added to the AuNH array, which in turn initiates the HCR. Au-AgNCs and Raman reporters aggregated on H3 to form long-stranded DNA, and the adjacent Au-AgNCs generated electromagnetic field enhancement effects. The quantitative detection of HR-HPV can be achieved by revealing the position and intensity of the Raman peaks, with an LOD of 0.76 pg/mL. The clinical blood samples were tested using the proposed method, and the results were consistent with PCR. Therefore, the development of CHA-HCR based SERS biosensor provides a potential ideal tool for early screening of HPV.

Enzyme-free nucleic acid amplification involves a long reaction time and complex sequence design or validation. Currently, there is no standard protocol for reference or rules for the design of each nucleotide.

## 5 CRISPR/Cas-related HR-HPV detection methods

**Figure 5:**
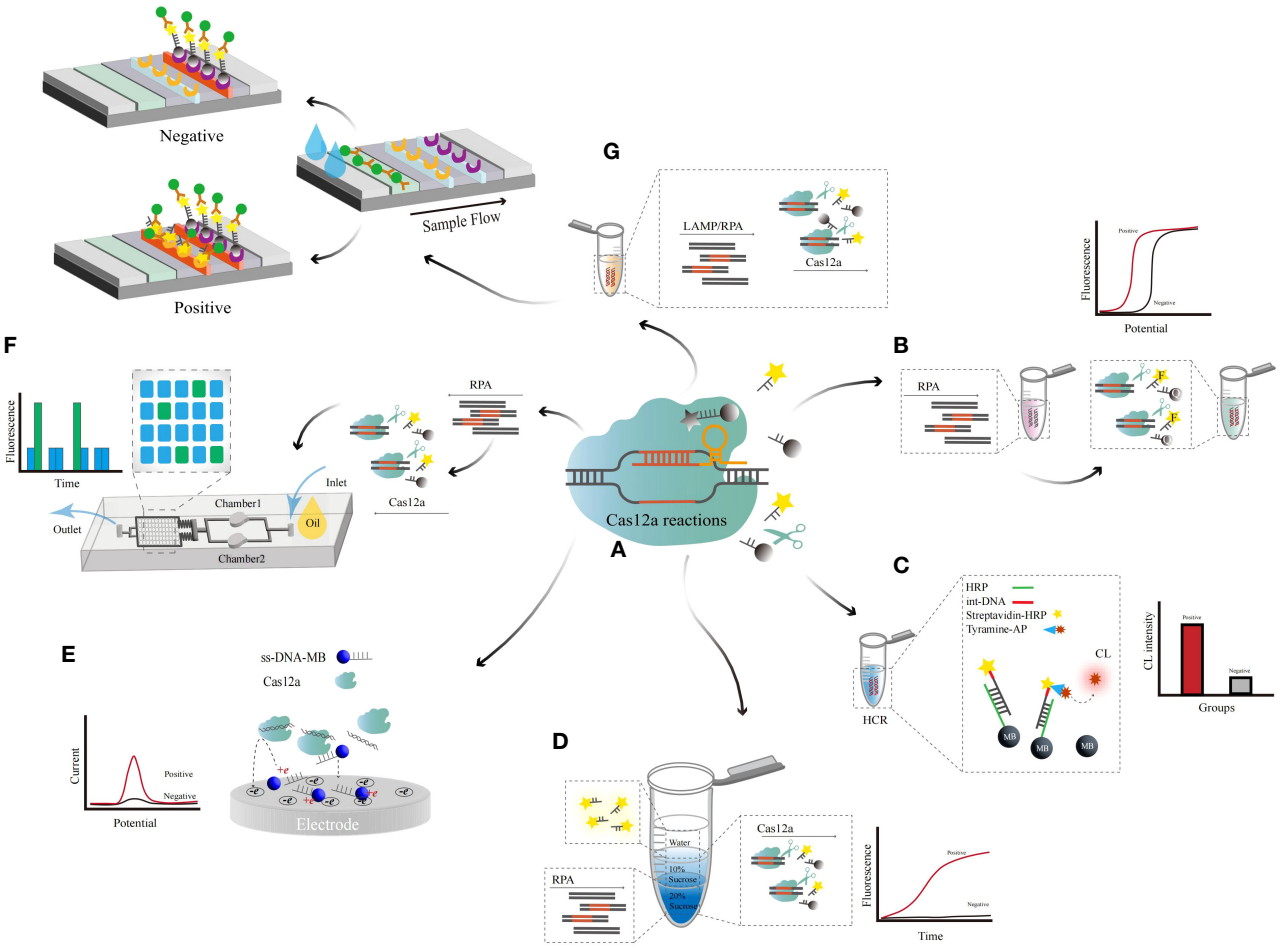
The principle of CRISPR/Cas12a-related detection. **(A)** Cas12a-based cleavage assay principle. **(B)** The Cas12a-based combined RPA amplification method was evaluated via a fluorescence reader. **(C)** The Cas12a-based chemiluminescence-enhanced biosensor. Cas12a cleaves amino-captured biotin DNA wrapped by HRP-recognizing MBs, and the large amount of tyrosine-AP attached to HRP causes a significant increase in the detection signal. **(D)** A dynamic multiple aqueous phase reaction detection system based on Cas12a. One-step detection was achieved by diffusion of the RPA reaction in the high-density liquid phase to the low-density liquid phase for Cas12a cleavage and triggering of the fluorescence signal **(E)** Of the Cas12a-based electrochemical biosensor. The trans-cleavage activity of activated Cas12a releases fewer single MB probes, resulting in an increased electrochemical current during detection **(F)** In the Cas12a-based microfluidic chip assay. The reaction solution of RPA and Cas12 was added to the chip, and the detection result was determined by reading the fluorescence value **(G)** Of the Cas12a-based LFA assay. Visualization of LFD detection by amplification of DNA via LAMP and the Cas12a effector cleavage reaction.

The clustered regularly interspaced short palindromic repeats (CRISPR/Cas) system, a revolutionary gene editing technique, has begun to be applied in the field of nucleic acid diagnosis (Wang et al., 2016). Currently, most CRISPR/Cas systems used in the field of HR-HPV detection use fluorescence detection for diagnosis (Larson et al., 2013; Broughton et al., 2020). Among them, CRISPR/Cas9 specifically recognizes and cleaves DNA by binding to ds-DNA with the assistance of a PAMmer (Gootenberg et al., 2018; Kellner et al., 2019). CRISPR/Cas12a can recognize and cleave target ds-DNA under CRISPR-RNA (cr-RNA) guidance (**Figure 5A**), exhibiting nonspecific cleavage activity against ss-DNA (Zhou et al., 2018; Gong et al., 2021; Zhang et al., 2021).

### 5.1 CRISPR/Cas9-related biosensors

CRISPR/Cas9 was utilized in combination with a bionic photonic crystal barcode detection platform (Zhang et al., 2021). The CRISPR/Cas9 system recognizes and cleaves DNA, and with the amplification of Cas9 and small guide RNA complex, Klenow fragment and strand displacement amplification, a large amount of ss-DNA can be generated. The photonic crystal barcode wrapped in polydopamine can capture ss-DNA, and the HCR can be triggered by the latter by FAM-labeled H1 and H2. By further amplifying the signal using HCR, multiple sequences of HR-HPV were detected simultaneously, with an LOD of 0.025 pM. The bio-inspired multi-system platform of HCR-CRISPR/Cas9 proved to be capable of simultaneous high sensitivity and multiplexed detection, which has great potential in clinical disease diagnosis.

### 5.2 CRISPR/Cas12a-related biosensors

Jennifer A. Doudna et al. integrated Cas12a and RPA techniques to detect HPV-16 and HPV-18 using fluorophore- and quencher-labeled ss-DNA (ss-DNA-FQ) as output signals (**Figure 5B**). Under the guidance of cr-RNAs, Cas12a can recognize and perform specific cleavage of the target ds-DNA (Chen et al., 2018), accompanied by nonspecific cleavage of ss-DNA. By combining CRISPR/Cas12a and RPA, a method for single detection of 13 types of HR-HPV can be achieved (Gong et al., 2021), with an LOD of 500 copies in a single reaction (35 min).

Hu, Tao et al. introduced a tyramine signal combined with CRISPR/Cas12a cleavage-triggered chemiluminescence (CL) to enhance the biosensor during the amplification process (Hu et al., 2022). MBs bind to hybrid strand DNA, which is composed of amino acid capture DNA and biotin recognition DNA (**Figure 5C**). CRISPR/Cas12a cleaves the target DNA into a short product, and the subsequent introduction of streptavidin-HPR and tyrosine-AP into the system results in a large number of tyrosine residues covalently conforming to the DNA, revealing enhanced chemiluminescence. The camera recorded the image, which showed an enhancement CL signal, with an LOD of 17 pM/single copy evaluated. In the same year, they set up the new CL-CRISPR platform by adding CL-enhanced biosensors to HCR and CRISPR (Ke et al., 2022). As above, when the target DNA is present, CRISPR/Cas12a cuts the target gene into short fragments, and incomplete DNA does not result in TMSD. The end of the hybrid chain DNA triggers the opening of hairpin structures (H1 and H2), which initiates the HCR to form a large number of long ds-DNA frameworks. The CCD camera directly detects the CL signal of large amounts of attached streptavidin-AP and is positively correlated with the target DNA concentration. The HR-HPV plasmid can achieve single copy per reaction detection, with an LOD of 3 pM.

A dynamic aqueous multiphase reaction system utilizing density contrast of sucrose concentration coupled with CRISPR/Cas12a was used to establish a diagnostic platform for HR-HPV (Yin et al., 2020) (**Figure 5D**). This miscible multiphase system supplies a unique dynamic diffusion interface that can combine incompatible but related reactions. With a high-density substrate, the DNA was amplified through an RPA reaction. The amplified products dynamically diffuse to the low-density top layer, triggering the nonspecific cutting activity of the Cas12a endonuclease. The fluorescent or quencher-labeled ss-DNA probe is further cleaved to generate a fluorescent signal. A 3D printing microfluidics device was constructed from black polylactic acid fibers, and the fluorescence was read directly under blue light. This method realizes multichannel and multidetection in a one-pot system with an LOD of 10-100 copies. The method can detect HPV human swab samples and plasma samples without the need for complex pretreatment. The sensitivity and accuracy of the assay are consistent with the PCR method.

Field potential-enhanced electric field-enhanced and CRISPR−;Cas12a electrochemical biosensors can be used to achieve target DNA detection in a homogeneous liquid phase (Li et al., 2021). Methylene blue-labeled ss-DNA (ss-DNA-MB) was used as the biosensor (**Figure 5E**). A pulsed electric field is applied to attract ss-DNA to the positively charged working electrode surface due to the static electric force. CRISPR/Cas12a is activated to cleave ss-DNA-MB, releasing methylene blue. The diffusion rate of the lysed methylene blue to the negative electrode surface and the decrease in the electrostatic repulsion force on the electrode surface led to an increase in the EC signal. By utilizing the difference in diffusion rates between electrochemical oligonucleotide probes and CRISPR cleavage probes on negatively charged working electrodes, a simple and sensitive DNA electrochemical detection was achieved without the need for complex electrochemical probe immobilization treatment. The peak redox current was measured by differential pulse voltammetry, with an LOD of 1 pM.

Combining CRISPR/Cas12a with a microfluidic double-drop device (M-D3) and a multi-RPA assay enables multipathway detection of HR-HPV (Zhao et al., 2023). M-D3 is made of polydimethylsiloxane, using standard soft lithography technology to prepare liquid drop devices and pressure and vacuum to control fluid technology (**Figure 5F**). This platform encapsulates specific target DNA and related CRISPR/Cas12a cr-RNAs into two sets of droplets. It triggers the nonspecific cleavage activity of Cas12a, generating corresponding fluorescence signals. HR-HPV was effectively detected based on the fluorescence intensity of the droplets. The reaction time was approximately 30 min, with an LOD of 10^-18^M. By optimizing LFAs, microfluidics and CRISPR/Cas12a, visualization of HR-HPV DNA can be achieved. A single-cluster, three-channel microfluidic device with microchannels and reaction chambers was designed using AutoCAD. RPA reagent, Cas12a and cr-RNA were added to the two associated chambers and incubated, after which the two solutions were mixed and reacted in the lysis chamber. Finally, the liquid is pulled into the LFA chamber to obtain a reading. The control line of the LFA was coated with streptavidin, and the detection line was coated with anti-FAM antibodies. The target DNA gene is cut into fragments, and the intact gene retained in the complex can be captured by streptavidin. Simultaneous detection of HPV-16 and HPV-18 DNA can be achieved under reaction conditions at 39 °C (30 min), with LODs of 10^-18^ M (approximately 1 copy/reaction) and 0.5 nM (Zhou et al., 2023). Firstly, RPA detection and Cas12a/crRNA incubation were performed, followed by lysis assay and readout. The human cervical swab sample were added directly to the chip heated for 12 min (39 °C), and then attracted to the lysis chamber through a pipette, syringe, or micropump. After the cracking experiment is completed, further pull the solution in the cracking chamber to the front end of the LFA, and finally read the results. The method was validated on real clinical samples and achieved 100% agreement compared to PCR methods. The method has great potential in screening HR-HPV and may also achieve patient subsampling self-inspection in the future, which will greatly alleviate the shortage of medical resources. Zhichen Xu et al. designed a starburst-shaped chip with 30 branches emitted from the center of the disk to the surrounding area (Xu et al., 2022) and expanded nine HR-HPV subtypes by RPA through fluorescent probes connecting a central inlet to 30 outlets. The outlet is preloaded with Cas12a, which can show the relevant target HPV subtypes, and the CRISPR-based lysis test is used for testing. The multichannel detection time was 40 min, with an LOD of 0.26 aM and an approximate length of 10^-17~18^ M. The consistency between CRISPR/Cas12a-RPA and PCR was 100%, and both HPV16 and HPV18 genotypes can be detected simultaneously within 40 min.

Mukama et al. designed a target DNA from the L1 gene of HR-HPV and designed an assay combining the CRISPR/Cas12a, LFAs and LAMP methods (Mukama et al., 2020) (**Figure 5G**). Cas12a cuts the LAMP product and the biotinylation ssDNA reporter, and lysis was monitored via the LFA biosensor. Biotin goat anti-mouse polyclonal IgG was immobilized on the control line, the ss-DNA probe was immobilized on the test line, and the AuNP-SA complex and intact ss-DNA gene were immobilized on the coupling pad. The Cas12a trans-cut ss-DNA reporter gene could not bind to the complementary DNA sequence fixed on the LFA test line. Single-copy sensitivity was achieved, with an LOD of 3.1×10^-21^ M (<1 copy). Compared the features of the RPA, RPA-LFA, RPA-Microfluidic, HCR-ECL, CHA-SERA and other methods in **Table 3** with the methods in **Table 1**, the new biosensors are almost always capable of highly sensitive, highly specific, rapid, and portable detection (**Table 3**).

**Table 3:** The performance comparison of other biosensors in HR-HPV detection.

Amplification Technology	Transduction	Times (min)	LOD	POCT	Cost	Instrument	Accuracy (%)	Specificity (%)	Sensitivity (%)	PPV (%)	NPV (%)	Ref.
RPA	LFA	60	10 copies	Yes	Low-cost	Portable	100	100	100	-*	-*	(Wang et al., 2020)
		60	100 copies/μL	Yes	Low-cost (8 dollars)	Portable	78	92.3	95.1	98.6	75.9	(Ma et al., 2019)
	Microfluidic	30	10 copies/μL	Yes	-*	Portable	100	100	100	100	100	(Cui et al., 2022)
	CRISPR/Cas12a	35	500 copies/reaction	Yes	Low-cost	Portable	-*	100	-*	100	100	(Gong et al., 2021)
	CRISPR/Cas12a- Microfluidic	40	10^-17~18^ M	Yes	Low-cost	Portable	-*	98.1	97.8	97.83	98.15	(Xu et al., 2022)
		30	10^-18^ M copy/reaction	Yes	Low-cost	Portable	97.5	100	92.3	100	96.43	(Zhao et al., 2023)
	CRISPR/Cas12a- Microfluidic-LFA	30	10^-18^ M	Yes	Low-cost (2 dollars)	Portable	-*	100	100	100	100	(Zhou et al., 2023)
	CRISPR/Cas12a-CL	120	17 pM/single copy	Yes	Low-cost	Portable	-*	100	87.50	100	92.31	(Hu et al., 2022)
	CRISPR−;Cas12a-EC	60	1 pM	Yes	Low-cost	Portable	-*	100	-*	-*	-*	(Li et al., 2021)
	CRISPR/Cas12a-dynamic aqueous multiphase reaction	60	10-100 copies	Yes	Low-cost	Portable	-*	100%	-*	-*	-*	(Yin et al., 2020)
HCR	ECL	60	0.01 pM	Yes	Low-cost	Heavy	97.9	100	100	-*	-*	(Wu et al., 2022)
		60	1.41 aM	No	Low-cost	Heavy	100	100	100	-*	-*	(Lu et al., 2022)
	CRISPR/Cas12a-CL	195	3 pM	Yes	Low-cost	Portable	100	100	88.89	88.89	100	(Ke et al., 2022)
	CRISPR/Cas9-Photonic crystal	30	0.025 pM	Yes	Low-cost	Portable	97.9	100	-*	-*	-*	(Zhang et al., 2021)
CHA	SERS	90	0.76 pg/mL	No	Low-cost	Heavy	96.3	100%	100%	100	100	(Dan et al., 2022)

NPV, Negative predictive value.

PPV, Positive predictive value.

-*, No test.

## 6 Future perspectives and conclusions

In this review, different HR-HPV detection methods adapted for use in research laboratories, both widely used and new methods, were summarized, and their benefits and limitations were discussed (Veyer et al., 2020) (**Table 1**). PCR possesses high sensitivity and specificity, and is the most common method for diagnosing HPV, but its drawbacks are quite obvious, it is difficult to conduct HPV diagnosis in a resource-constrained environment. Moreover, mass screening is not suitable for use in developing countries or areas with limited economic access. Therefore, the development and application of new methods are important tools for complementing and enabling global human papillomavirus screening. Compared to traditional PCR, q-PCR reduces clinical false-positives with high sensitivity. dd-PCR is able to detect HR-HPV in plasma with nanoscale droplets generated, showing significantly greater sensitivity than q-PCR (G. and Helenius, 2017). However, thermocycling is still needed to separate the two DNA strands, which means that the precise control of temperature changes is needed together with specialized technicians. At present, PCR amplification of nucleic acids is still recommended. In the future, if the shortcomings of nonportable and complex operations can be addressed, there will be more widespread applications in the area of rapid molecular diagnosis.

LAMP and RPA are emerging isothermal amplification technologies that are simple alternative technologies for rapid and portable detection. These methods have great potential in POCT, but there are still some shortcomings. First, all of these methods require other operations to display the amplification results. LFAs can be used in conjunction with other methods, such as direct visual readout methods through the use of a labeled probe and reverse primer. In addition, the RPA assay can be coupled with nanoparticle-based methods because of its high specificity (Wang et al., 2020). Therefore, combination with LFA could effectively overcome the shortcomings caused by complex operation. Second, all of these methods suffer from false-positive results due to aerosol contamination during nucleic acid amplification. A microfluidic self-digitizing chip constructed through a 3D printer has a high-temperature resistant and sealed liquid storage structure (Cui et al., 2022). Furthermore, multiplexing chip channels can simultaneously achieve multigenotype HR-HPV detection in one sample (Wang et al., 2021; Mei et al., 2023). After investing a certain amount of primer design cost, the production price of microfluidic chips can also be controlled to be low. At the low-resource areas in the future, this may be an HR-HPV screening method. Thus, the combination of microfluidics with LAMP and RPA could efficiently eliminate false-positivepositives. Finally, they are dependent on enzyme activity. Therefore, enzyme-free amplification has the advantages of great biocompatibility, isothermal conditions and excellent amplification efficiency. Due to its unique amplification products, it is easy to generate excessive byproducts, resulting in excessively high background signals (Bi et al., 2017). ECL methods have a low background, portable instruments, and easy operation, which significantly increase sensitivity (Dan et al., 2022; Lu et al., 2022; Wu et al., 2022). However, the nucleic acid amplification process and detection process are still divided into two steps. If they can be integrated into a one pot approach, it will be helpful for the application of POCT. EC catalysts that combine nucleic acids with noble metal nanostructures such as ZnO nanoparticles, AuNPs and AuNH promote electron transfer and enhance ECL signaling. Nanomaterials not only are nontoxic and harmless but also have stable performance and easy surface functionalization, allowing them to be used as natural and efficient biosensors. It has potential application value in various biosensing and clinical applications, as well as other biochemical analyses. The use of ECL sensors to measure human serum samples shows that the biosensor has high accuracy. However, both isothermal amplification and enzyme-free amplification are limited by the strict primer design and lack of primer design software or tools. LAMP and RPA require four to six primers and enzymes. Moreover, HCR and CHA require primers containing H1, H2 and initiator I, which are more complex than isothermal amplification. If the primer design problem can be solved, significant progress in the field of rapid detection and screening of cervical cancer could occur.

CRISPR-based technology has been widely used in the field of molecular diagnosis because it involves rapid, highly sensitive reactions (Zhou et al., 2018; Kellner et al., 2019; Broughton et al., 2020). However, the processing of samples is complex and typically requires separate operations for pretreatment and nucleic acid amplification. As we summarize the NA-based detection methods, they are also all based on the basic principle of amplifying HPV nucleic acid sequences, so it is only necessary to design reasonable primers according to different types of HPV (regular HPV or HR-HPV) to achieve rapid detection. The reaction system can be integrated into a closed space using microfluidic chips to achieve one-step detection and can also avoid environmental pollution caused by the generation of aerosols. If these issues can be resolved, CRISPR/Cas12a-LFA will become a great method for large-scale screening of HPV. Moreover, integrating LFAs into microfluidic chips enables portable and intuitive detection (Zhao et al., 2023; Zhou et al., 2023), a slight negative pressure is applied to the unit to ensure an absolute seal and reduce the generation of cross-contamination. Nucleic acid amplification and Cas12a/cr-RNA incubation were first carried out, followed by cleavage and readout assays. The integrated system shortens the procedure, improves efficiency, and avoids sample transfer, thus reducing potential contamination and false-positive results. Although the entire system requires a long time to design and develop, if mass production can be realized, it will be a major application in molecular diagnostics and POCTs for HR-HPV.

To summarize, nucleic acid testing for HR-HPV is essential for screening and aiding in the control of cervical cancer (De Sanjose et al., 2018; Perkins et al., 2023). In the future, more rapid, sensitive, and portable tests will not only support HPV screening but also help in the early diagnosis of cervical cancer. Traditional methods have the advantages of stability and high reproducibility but are being gradually phased out due to cumbersome detection methods. Research on HR-HPV detection will mainly focus on the development of multiplex high-throughput biosensors, one-step biosensors, precious metal nanomaterials, and extremely sensitive detection methods to adapt POCT and companion diagnostics for developing countries, impoverished areas and other increasingly complex environments.

## Author contributions

CM: Conceptualization, Investigation, Methodology, Visualization, Writing – original draft. MZ: Investigation, Methodology, Visualization, Writing – review & editing. NX: Visualization, Writing – review & editing. YL: Conceptualization, Funding acquisition, Methodology, Supervision, Visualization, Writing – review & editing. YW: Conceptualization, Methodology, Supervision, Writing – review & editing.
